# Supplementary material for: Human muscle activity and lower limb biomechanics of overground walking at varying levels of simulated reduced gravity and gait speeds
Source: PLoS One. 2021 Jul 14;16(7):e0253467. doi: 10.1371/journal.pone.0253467 (PMC8279339; doi:10.1371/journal.pone.0253467)
Supplement: S1 Table — (DOCX) [file pone.0253467.s001.docx]

S1 Table: The unadjusted p-values of the effects of simulated gravity level (Gravity), walking speed (Speed), and the interaction between walking speed and gravity level on dependent variables.

|  | |  | **p value** |  |
| --- | --- | --- | --- | --- |
| **Variable** | | **Gravity** | **Speed** | **Interaction**  **(Speed*Gravity)** |
| Force | Maximum vertical | <0.001 | <0.001 | <0.001 |
|  | Maximum braking | <0.001 | <0.001 | 0.147 |
|  | Maximum accelerating | <0.001 | <0.001 | <0.001 |
|  | Maximum absolute medial-lateral | <0.001 | <0.001 | <0.001 |
| Moment | Hip flexion | <0.001 | <0.001 | <0.001 |
|  | Knee extension (early-mid stance) | <0.001 | <0.001 | <0.001 |
|  | Knee extension (transition) | 0.046 | <0.001 | 0.566 |
|  | Ankle plantarflexion | <0.001 | <0.001 | <0.001 |
| Power | Hip generation | <0.001 | <0.001 | <0.001 |
|  | Knee generation (early-mid stance) | <0.001 | <0.001 | <0.001 |
|  | Knee absorption (transition) | <0.001 | <0.001 | <0.001 |
|  | Ankle generation | <0.001 | <0.001 | <0.001 |
| Stance phase RMS EMG | Rectus femoris | <0.001 | 0.013 | 0.419 |
|  | Vastus lateralis | <0.001 | <0.001 | 0.010 |
|  | Vastus medialis | <0.001 | <0.001 | 0.004 |
|  | Biceps femoris | <0.001 | <0.001 | <0.001 |
|  | Lateral gastrocnemius | 0.003 | <0.001 | 0.942 |
|  | Medial gastrocnemius | <0.001 | <0.001 | 0.888 |
|  | Soleus | 0.250 | <0.001 | 0.268 |
|  | Tibialis anterior | 0.093 | <0.001 | 0.016 |
| Swing phase RMS EMG | Rectus femoris | 0.740 | <0.001 | 0.397 |
|  | Vastus lateralis | 0.524 | <0.001 | 0.262 |
|  | Vastus medialis | 0.885 | <0.001 | 0.035 |
|  | Biceps femoris | 0.090 | <0.001 | 0.811 |
|  | Tibialis anterior | <0.001 | <0.001 | 0.099 |
| Spatial | Right stride length | 0.306 | <0.001 | 0.430 |
