# Supplementary material for: Human muscle activity and lower limb biomechanics of overground walking at varying levels of simulated reduced gravity and gait speeds
Source: PLoS One. 2021 Jul 14;16(7):e0253467. doi: 10.1371/journal.pone.0253467 (PMC8279339; doi:10.1371/journal.pone.0253467)
Supplement: S2 Table — Table shows the number of participants for whom data is missing. No number means no data were missing for that condition and data type. The abbreviated headings of the columns are: tibailis anterior (TA), soleus (Sol), medial gastrocnemius (MGas), lateral gastrocnemius (MGas), rectus femoris (RecF), vastus medialis (VM), vastus lateralis (VL), and biceps femoris (BF). (DOCX) [file pone.0253467.s002.docx]

**S2 Table.** Missing data in conditions. Table shows the number of participants for whom data is missing. No number means no data were missing for that condition and data type. The abbreviated headings of the columns are: tibailis anterior (TA), soleus (Sol), medial gastrocnemius (MGas), lateral gastrocnemius (MGas), rectus femoris (RecF), vastus medialis (VM), vastus lateralis (VL), and biceps femoris (BF).

| **Speed** | **Simulated Gravity** | **TA** | **Sol** | **MGas** | **LGas** | **RecF** | **VasM** | **VasL** | **BicF** | **Force** |
| --- | --- | --- | --- | --- | --- | --- | --- | --- | --- | --- |
| 0.4 ms^-1^ | 1 G |  |  |  |  |  |  | 1 | 1 |  |
|  | 0.76 G |  |  |  |  |  |  | 1 |  |  |
|  | 0.45 G |  |  |  |  |  |  | 1 |  |  |
|  | 0.31 G |  |  |  |  |  |  | 1 |  |  |
| 0.8 ms^-1^ | 1 G |  |  |  |  |  |  | 3 |  |  |
|  | 0.76 G |  |  |  |  |  | 1 | 2 |  |  |
|  | 0.45 G |  |  |  |  |  |  | 1 |  |  |
|  | 0.31 G |  | 1 |  |  |  |  | 2 |  |  |
| 1.2 ms^-1^ | 1 G | 1 | 1 | 1 | 1 | 1 | 1 | 2 | 1 | 1 |
|  | 0.76 G |  |  |  |  | 1 |  | 1 |  |  |
|  | 0.45 G |  |  |  |  |  |  | 1 |  |  |
|  | 0.31 G |  |  |  |  |  |  | 1 |  |  |
| 1.6 ms^-1^ | 1 G |  |  |  |  |  |  | 1 |  |  |
|  | 0.76 G |  |  |  |  |  |  | 1 |  |  |
|  | 0.45 G |  |  |  |  |  |  | 1 |  |  |
|  | 0.31 G | 2 | 2 | 2 | 2 | 2 | 2 | 3 | 2 | 2 |
