# Supplementary material for: Human muscle activity and lower limb biomechanics of overground walking at varying levels of simulated reduced gravity and gait speeds
Source: PLoS One. 2021 Jul 14;16(7):e0253467. doi: 10.1371/journal.pone.0253467 (PMC8279339; doi:10.1371/journal.pone.0253467)
Supplement: Attachment — Submitted filename: ReviewerComment_PLOS.docx [file pone.0253467.s004.docx]

1. Please ensure that your manuscript meets PLOS ONE's style requirements, including those for file naming. The PLOS ONE style templates can be found at

[https://journals.plos.org/plosone/s/file?id=wjVg/PLOSOne_formatting_sample_main_body.pdf](https://urldefense.proofpoint.com/v2/url?u=https-3A__journals.plos.org_plosone_s_file-3Fid-3DwjVg_PLOSOne-5Fformatting-5Fsample-5Fmain-5Fbody.pdf&d=DwQGaQ&c=sJ6xIWYx-zLMB3EPkvcnVg&r=JnnS7Ct7wSh3pzPHN32B9ktLLCw8jk68Po03ZI_XkIg&m=Mfa9-7wan9uNSdSLKisMLE5RqO4dKSmP2nnvnae0y-Y&s=gQ3_SR87PO7MkW_xUOks6toCyB9YTKbyXi59WCaddyQ&e=) and

[https://journals.plos.org/plosone/s/file?id=ba62/PLOSOne_formatting_sample_title_authors_affiliations.pdf](about:blank)

Changes made as requested.

2. Please correct your reference to "p=0.000" to "p<0.001" or as similarly appropriate, as p values cannot equal zero.

Thank you for pointing this out. The p values have been corrected throughout.

3**.In your Data Availability statement, you have not specified where the minimal data set underlying the results described in your manuscript can be found. PLOS defines a study's minimal data set as the underlying data used to reach the conclusions drawn in the manuscript and any additional data required to replicate the reported study findings in their entirety. All PLOS journals require that the minimal data set be made fully available.** For more information about our data policy, please see [http://journals.plos.org/plosone/s/data-availability](https://urldefense.proofpoint.com/v2/url?u=http-3A__journals.plos.org_plosone_s_data-2Davailability&d=DwQGaQ&c=sJ6xIWYx-zLMB3EPkvcnVg&r=JnnS7Ct7wSh3pzPHN32B9ktLLCw8jk68Po03ZI_XkIg&m=Mfa9-7wan9uNSdSLKisMLE5RqO4dKSmP2nnvnae0y-Y&s=5cE1cBien5NdL8DjewskvchrOCaiu6llbhTPsqbm4rE&e=).

Upon re-submitting your revised manuscript, please upload your study’s minimal underlying data set as either Supporting Information files or to a stable, public repository and include the relevant URLs, DOIs, or accession numbers within your revised cover letter. For a list of acceptable repositories, please see [http://journals.plos.org/plosone/s/data-availability#loc-recommended-repositories](https://urldefense.proofpoint.com/v2/url?u=http-3A__journals.plos.org_plosone_s_data-2Davailability-23loc-2Drecommended-2Drepositories&d=DwQGaQ&c=sJ6xIWYx-zLMB3EPkvcnVg&r=JnnS7Ct7wSh3pzPHN32B9ktLLCw8jk68Po03ZI_XkIg&m=Mfa9-7wan9uNSdSLKisMLE5RqO4dKSmP2nnvnae0y-Y&s=J5gCWqDBiGskK6gQATQ5GgZNoAuaO3Ka3G3YJ4Kl-Fs&e=). Any potentially identifying patient information must be fully anonymized.

Important: If there are ethical or legal restrictions to sharing your data publicly, please explain these restrictions in detail. Please see our guidelines for more information on what we consider unacceptable restrictions to publicly sharing data: [http://journals.plos.org/plosone/s/data-availability#loc-unacceptable-data-access-restrictions](https://urldefense.proofpoint.com/v2/url?u=http-3A__journals.plos.org_plosone_s_data-2Davailability-23loc-2Dunacceptable-2Ddata-2Daccess-2Drestrictions&d=DwQGaQ&c=sJ6xIWYx-zLMB3EPkvcnVg&r=JnnS7Ct7wSh3pzPHN32B9ktLLCw8jk68Po03ZI_XkIg&m=Mfa9-7wan9uNSdSLKisMLE5RqO4dKSmP2nnvnae0y-Y&s=h18Q3Bhy26Hr8WnT1xvfggTJlIkL8a2UvhgWr0EjqMA&e=). Note that it is not acceptable for the authors to be the sole named individuals responsible for ensuring data access.

We will update your Data Availability statement to reflect the information you provide in your cover letter.

We have updated the data availability information in the manuscript.

4. Please include a caption for figure 10.

A caption has been included for Figure 10.

5. Please include captions for your Supporting Information files at the end of your manuscript, and update any in-text citations to match accordingly. Please see our Supporting Information guidelines for more information: [http://journals.plos.org/plosone/s/supporting-information](about:blank).

We have provided captions and updated the in-text citations to S1 video and S2 table.

Reviewer #1:

Dear Author,

This is a well written and comprehensive paper. The rationale and discussion are clear and concise. Thank you.
I have a few minor comments that would help the reader.
Thank you for your feedback and comments on the paper. We appreciate your time.

**General comments:**
It would be useful to include a brief description of the marker set, muscles studied, and EMG placement in the methods. As well as a description of the method used to calculate the hip joint centres, the equations used to calculate the anthropometric model, and the coordinate system of segments.
See a recent publication by Derrick et al. ([https://doi.org/10.1016/j.jbiomech.2019.109533](https://urldefense.proofpoint.com/v2/url?u=https-3A__doi.org_10.1016_j.jbiomech.2019.109533&d=DwQGaQ&c=sJ6xIWYx-zLMB3EPkvcnVg&r=JnnS7Ct7wSh3pzPHN32B9ktLLCw8jk68Po03ZI_XkIg&m=Mfa9-7wan9uNSdSLKisMLE5RqO4dKSmP2nnvnae0y-Y&s=y8r1N9cpW3mgLnz71eAoovS2xbpnt-eVMNMQzCBdc_g&e=))
We have included a short description of the marker set and the EMG placement in the Data Collection sub-section in the methods.

Details on the anthropometric model have also been included and a reference to Visual3D marker set guide and model building guide have been incorporated.

**Specific comments:**
*Line 14: Replace "gait biomechanics" with "lower limb biomechanics".*
Change made as suggested.

*Line 153: Should it be stride length instead of step length?*
It absolutely should. Thank-you for catching this error. We have corrected the mistake.

Line 158-159: Replace semi-colons with commas.

Grammar has been corrected.

Line 302-302: Do you mean "The benefit of smaller ground reaction force magnitude is reduced lower limb joint loading during walking?" Using lower limb instead of reduced lower limb can be confusing for the reader as you refer to lower limb biomechanics, and "gait" is a pattern of locomotion rather than an activity.

You are correct. Thank you for pointing out the confusing English in this sentence and for suggesting better phrasing.

Line: 353: Consider rephrasing to remove the use of "likely" twice in the same sentence.
Thank you for improving the readability of the paper.

Line 407: Include a space between reference and text.
Thank you for catching this. We have corrected the error.

Reviewer #2:

This is an interesting study on the effects of simulated reduced gravity on walking of healthy young participants. In a factorial design, the effects of weight support and speed on a number of relevant variables are assessed. I have read the manuscript with great interest, the research question seems relevant, and the study seems well conducted. My main comments concern the analysis and discussion section. I have further specified my concerns below.
Thank you for your time and detailed feedback. We hope that we have addressed all of your comments to a satisfactory level and appreciate your suggestions for improving our manuscript.

**Main comments**
The authors have chosen to vary weight support over a wide range. In addition, all participants walked at 4 different speeds, ultimately resulting in a factorial design with 16 cells. The assumption that underlies this design is that the effects of reduced gravity vary as a function of walking speed. However, the statistical analysis and, to some extent, the discussion section, focus mainly on the effects of reduced gravity rather than the interaction between reduced gravity and walking speed. Nevertheless, the figures seem to indicate that such interactions do exist for certain parameters. These interactions may be relevant e.g. for clinical application in patients with very slow walking speeds. So I wonder whether it would be useful to include the interactions in the statistical model and present the results in the manuscript (possibly as supplementary material?).
In this manuscript, we are focusing on the effect of gravity. Although it would be interesting and relevant to investigate the interaction between speed and gravity, it is not particularly feasible in this paper. We evaluate the significance of gravity on 26 dependent variables. We do not have enough participants to investigate the interaction effect on the 26 dependent variables – it would severely underpower the study. Presentation and interpretation of the interaction effect would also further increase the length of this manuscript. We have included the interaction effects as Supplementary Table 1 as requested.

The discussion sometimes seems unnecessarily long. In certain passages, an extensive literature review is given with respect to a particular result without making much of a point about the data collected in the current study. I think the discussion can have more impact if it would be slightly more concise.

And

at some points the discussion can be a bit more concise. E.g. the comparison with previous studies is relevant, but could perhaps be a bit more to-the-point. Some passages also seem to be a bit off-topic in the context of the present research question, e.g. a discussion of the reflex pathways.

To shorten the discussion and focus more on our own findings, we have added a table summarizing the findings of other articles. Although the table is rather large, this cuts down the size of the discussion substantially and improves the flow of the discussion.

**Introduction**
Lines 62-65: It may be worth specifying here why and how walking speed may affect the effects of reduced gravity (and make clear why it would be interesting to study the interactions between both variables).
We have added a statement explaining how walking speed affects muscle recruitment. We have also included Supplementary Table 1 to address the interactions effects between speed and gravity level.

I appreciate that the authors take the trouble to formulate a number hypotheses. However, given the large number of variables, the hypotheses put forward seem somewhat arbitrary. As a reader I would have no qualms if this was presented as a descriptive study mapping (without specific hypotheses) the combined effects of speed and weight support .
Thank you for the comment and appreciation of our effort. The hypotheses mentioned in the introduction were the hypotheses we formed when designing this study. In deference to the scientific method, we feel that the hypotheses need to be included rather than modifying the hypotheses *post hoc*.

**Methods**
Because participants had to step on force plates, were the participants instructed in a specific way prior to the measurement (for example, were they instructed to walk ‘as normally as possible’ or to land on the heel)? This seems particularly relevant as reduced gravity at higher walking speed appears to result in a more dorsiflexed landing (see fig 4), which seems a bit counterintuitive.
The only instructions to the subjects were in regard to having an aerial phase during the trials. We defined walking to the subjects as bipedal gait without a flight phase and asked that participants not include a flight phase in any of the trials. This is now included in the manuscript.

Line 144: Was there initial heel contact in all conditions, or did high levels of reduced gravity also result in forefoot landing? If the latter is the case, initial contact seems to be a better term here than heel strike.

Thank you for raising this interesting point. I looked into how participants struck the ground using the motion capture data. I subtracted the vertical position of the Heel marker from the Toe marker, to determine if the toe was above or below the heel at initial contact. (I also subtracted the vertical offset between the markers in the static calibration pose, to ensure the result was an accurate indication of foot orientation). At initial contact in all trials the heel marker was below the toe marker, suggesting initial heel contact in all conditions.

We have updated the text to provide clarification that all initial contacts were heel strikes.

Lines 161-164: Why were the muscle data low-pass filtered after the time normalization and averaging? It is common to do this on the rectified, raw signal.

The purpose of the moving average window was for visual comparison between conditions in the figures. The data used for muscle RMS processing and statistical comparisons were the high pass rectified signals without any low pass filtering or inclusion of a moving average window. The full original data are included in the data repository.

Lines 173-175: Both speed and reduced gravity are included as factors in the statistical analysis. The main effects of speed (less relevant) and the interactions of speed with the level of gravity (certainly relevant) are not explicitly mentioned anywhere. Furthermore, it is not entirely clear to me what exactly the post hoc comparisons relate to. The different levels of the independent variable are not compared post hoc. Given the large number of dependent variables, some correction for multiple testing seems warranted, but I don't think these are ‘post hoc tests’.
We have updated the manuscript to refer to the Benjamini-Hochberg procedure as an adjustment for multiple comparisons.

**Results**
Line 181: 'some selected data points'. Is it possible to be a little more specific?

We have included a supplementary table to show all missing data.

Lines 181-183: How was this determined?

We determined the lifting force in a separate paper with a subset of the participants (MacLean & Ferris, 2020). Briefly, we implemented a load cell in the bodyweight support system to measure system force. We placed markers on the bodyweight support system to determine orientation and position relative to the participant’s pelvis (which we tracked with motion capture). We transformed the load cell force into vertical, medial, and anterior force on the participant. The force fluctuation increased with speed and bodyweight support level, but remained within a 10% margin.

I find the tables a bit confusing. The descriptive statistics are given for all variables in Table 1 and but not in Table 2. Perhaps the type of information provided in both tables can be it more consistent.

Table 1 is the descriptive data of the ground reaction forces. We have added headings and slightly modified the description to better explain the data in table 1. Table 2 shows the p-values from all statistical tests and does not describe the data. The description of the data used for the statistical tests is illustrated in Table 1, figure 7 and figure 9. Because the purpose of each table is different and the fundamental variables are different, we could not figure out how to improve consistency across all the tables.

With regard to Table 2: I don’t think you need to report the ranks and the results for the original test (before the Benjamini-Hochberg correction). Also, p-values of 0.000 are meaningless, please report p<.001.

The p-values of 0.000 have been corrected and the rank and original p-value columns have been removed. The table should be clearer to read and understand now. Thank you for catching this.

Some figures seem to be slightly redundant (in particular figures 3, 7, and 9) as they contain information that is already presented in other figures.

We understand your concerns. Figure 3 contains information in figure 2, but it combines data from 2 of the plots of figure 1 and isolates the points of interest. We found it was a good way of visualizing the changes in GRF profile without overwhelming the reader. The data in figure 7 is to assist with visualizing the data used in the statistical comparison. Although the data could be extracted from figures 4,5,&6, we thought it would be convenient for readers to have the peak data clearly displayed. Although figure 8 shows the time series data, we thought it was useful to have the RMS data represented in the spider plots.

Lines 251-255: I am not sure if this belongs in a Results section.

We removed it as requested.

Lines 255-256: These and similar passages suggest that subphases of the gait cycle have been analysed, but this is not the case if I understand correctly.

The RMS of muscle activity were analysed separately for stance phase and swing phase of the gait cycle. The data processing section has been updated to clarify this processing method. Table 2 and Figure 9 present the results of the stance and swing phase individually.

**Discussion**
General: much of the discussion focusses on the EMG, and I think the relationship between the kinetic/kinematic/neuromuscular variables could be discussed more thoroughly. The effects on, for example, the braking and propulsive impulse are likely to alter neuromuscular control. Furthermore, it is perhaps good to discuss where / how reduced gravity does or does not result in a breakdown of the normal locomotor pattern. This can be particularly relevant for clinical applications when therapists strive to impose a 'physiological' gait pattern (under conditions of reduced gravity). A related relevant point is whether or not reduced gravity results in changes in the underlying locomotor task. Although weight support in patients is intended to simplify the walking task (by reducing task demands associated with weight gain and stability) anyone who has ever walked with a lot of weight support (e.g. > 50%) knows that this is hard and fatiguing**.**

And

I do not find the paragraph on the clinical implications of the results very convincing. The main reason for having patients walk with reduced gravity (treadmill or overground) is to reduce problems in support and balance. In the context of motor (re-) learning it is important to know whether or not reductions in gravity result in deviating kinematic / kinetic / EMG patterns, in other words: does the weight support not introduce new task elements that are irrelevant for natural, overground walking, and does the support not result in a less ‘physiological’ gait pattern?

All of our participants reported that it was easy to learn and walk with the bodyweight support. Participants did comment that wearing the harness for hours at a time could become uncomfortable. Some participants reported that walking at the fast speed in 0.31 G would have been easier with a flight phase (i.e. loping). However, all subjects felt like it was easy to adapt to walking in the simulated reduced gravity. We found no fundamental changes to the locomotor pattern in kinematics or kinetics or EMG. Although amplitudes changed, the patterns remained fairly consistent. The response of participants and our data lead us to believe that subjects did not drastically alter neuromuscular control. We have added a paragraph discussing these implications in the discussion.

Lines 282-284: I am not convinced that the Soleus and Lateral Gastrocnemius show no effect of reduced gravity. In particular at the end of the stance phase at high speeds, there seems to be an effect. Because the statistical analysis of the EMG is based on RMS values calculated over the entire gait cycle, these effects may not have been detected. However, they do become clear in Figure 8.
The RMS values were found in stance and swing phase separately. The top row of figure 9 shows the RMS in the stance phase. We agree that there are visible effects of gravity at 0.4 m/s and 1.6 m/s. However, there is very little difference in the RMS at 0.8 and 1.2 m/s. The p values of effect of gravity in stance phase are 0.25 and 0.093 for the lateral gastrocnemius and soleus respectively.

Lines 286-288: This seems like a strange hypothesis, as the quadriceps contribute to weight acceptance during the early stance phase. So it seems logical that with reduced gravity the amplitude of the activity in this muscle group decreases.
We based our hypothesis from previous work by the 2^nd^ author of this paper, who found vastus lateralis activity did not change with simulated reduced gravity level (Ferris et al. 2001). In the discussion, we go into detail about this finding and compare it to past studies.

Lines 319-320: If you look at Figure 8, however, you can see that in the RF during the stance-swing transition, there appears to be an effect of reduced gravity.
The RF activity does decrease around the stand-swing transition with gravity in the 1.2 and 1.6 m/s walking conditions. However, there is no change in RF activity at the stance-swing transition in 0.4 and 0.8 m/s walking. Overall, we found the effect of gravity to not have a significant effect on RF activity in swing phase. A comparison of the RMS during the transition period may have statistical significance or present an interesting finding, but is not included in the scope of this manuscript.

Lines 352-354: Can the authors clarify this further? How (!) do differences in speed and the support system explain the discrepancies with previous studies that are observed?
and

Lines 416-420: I understand this is difficult to explain, but if differences between treadmill and overground are an explanation, this should be explained further: why? The effects are quite pronounced.

We have added a paragraph to address how differences in support system, speed, and walking environment can (at least partially) explain the differences.

Lines 356-358: I don't quite understand what the authors mean. Reformulate?
This sentence has been removed and the topic discussed in a dedicated paragraph in the discussion.

Lines 393-396: I find the suggestion interesting but perhaps the authors can further specify why / how conditions of reduced gravity changes the force-length relationship. Furthermore, the authors may be able to say something about why effects were in fact found in the MG.
We have updated the manuscript to include discussion on how the medial gastroc., lateral gastroc., and soleus respond differently to altered mechanical demand at the ankle.

We mention the force-length relationship as we believe it is relevant, but do not have the toolset to fully investigate this relationship in this manuscript. We are planning to investigate how gravity affects the force-length-velocity relationship during stance phase in a subsequent manuscript.

**Missing Conditions -** Table shows the number of participants for whom data are missing. No number means no data were missing for that condition and data type. The abbreviated headings of the columns are: tibailis anterior (TA), soleus (Sol), medial gastrocnemius (MGas), lateral gastrocnemius (MGas), rectus femoris (RecF), vastus medialis (VM), vastus lateralis (VL), and biceps femoris (BF).

| **Speed** | **Simulated Gravity** | **TA** | **Sol** | **MGas** | **LGas** | **RecF** | **VasM** | **VasL** | **BicF** | **Force** |
| --- | --- | --- | --- | --- | --- | --- | --- | --- | --- | --- |
| 0.4 ms^-1^ | 1 G |  |  |  |  |  |  | 1 | 1 |  |
|  | 0.76 G |  |  |  |  |  |  | 1 |  |  |
|  | 0.45 G |  |  |  |  |  |  | 1 |  |  |
|  | 0.31 G |  |  |  |  |  |  | 1 |  |  |
| 0.8 ms^-1^ | 1 G |  |  |  |  |  |  | 3 |  |  |
|  | 0.76 G |  |  |  |  |  | 1 | 2 |  |  |
|  | 0.45 G |  |  |  |  |  |  | 1 |  |  |
|  | 0.31 G |  | 1 |  |  |  |  | 2 |  |  |
| 1.2 ms^-1^ | 1 G | 1 | 1 | 1 | 1 | 1 | 1 | 2 | 1 | 1 |
|  | 0.76 G |  |  |  |  | 1 |  | 1 |  |  |
|  | 0.45 G |  |  |  |  |  |  | 1 |  |  |
|  | 0.31 G |  |  |  |  |  |  | 1 |  |  |
| 1.6 ms^-1^ | 1 G |  |  |  |  |  |  | 1 |  |  |
|  | 0.76 G |  |  |  |  |  |  | 1 |  |  |
|  | 0.45 G |  |  |  |  |  |  | 1 |  |  |
|  | 0.31 G | 2 | 2 | 2 | 2 | 2 | 2 | 3 | 2 | 2 |
